# Synergistic Manipulation of Na^+^ Flux and Surface‐Preferred Effect Enabling High‐Areal‐Capacity and Dendrite‐Free Sodium Metal Battery

**DOI:** 10.1002/advs.202103845

**Published:** 2022-01-09

**Authors:** Qianzheng Jin, Hongfei Lu, Zili Zhang, Jing Xu, Bin Sun, Yang Jin, Kai Jiang

**Affiliations:** ^1^ School of Electrical Engineering Zhengzhou University Zhengzhou 450001 P. R. China; ^2^ State Key Laboratory of Advanced Electromagnetic Engineering and Technology School of Electrical and Electronic Engineering Huazhong University of Science and Technology Wuhan 430074 P. R. China

**Keywords:** anode, high areal capacity, ion flux, metallic sodium batteries, surface‐preferred

## Abstract

The propensity of sodium anode to form uniform electrodeposit is bound up with the nature of electrode surface and regulation of Na‐ion flux, as well as distribution of electronic field, which is quite crucial for high‐areal‐capacity sodium metal batteries (SMBs). Herein, a novel metallic sodium/sodium–tin alloy foil anode (Na/NaSn) with 3D interpenetrated network and porous structure is prepared through facile alloy reaction. The strong sodiophilic properties of sodium–tin alloy can lower the nucleation energy, resulting in smaller depositing potential and strong adsorption of Na^+^, while synergistic effect of porous skeleton and additional potential difference (≈0.1 V) between Na and Na–Sn alloy (Na_15_Sn_4_) can alleviate volume expansion, redistribute the Na‐ion flux and regulate electronic field, which favors and improves homogeneous Na deposition. The as‐fabricated Na/NaSn electrode can endow excellent plating/stripping reversibility at high areal capacity (over 1600 h for 4 mAh cm^−2^ at 1 mA cm^−2^ and 2 mAh cm^−2^ at 2 mA cm^−2^), fast electrochemical kinetics (500 h under 4 mAh cm^−2^ at 4 mA cm^−2^) and superior rate performances. A novel strategy in the design of high‐performance Na anodes for large‐scale energy storage is provided.

## Graphical abstract

An Na anode integrating Na_15_Sn_4_ and metallic Na is prepared through facile alloy reaction between Na and Sn, enabling superior rate performances and uniform Na deposition under high capacity.

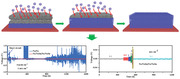

## 1 Introduction

Sodium ion batteries (SIBs) have been considered as a promising alternative to lithium ion batteries (LIBs) to integrate reproducible energy sources for scale stationary grids owing to the advantages of low cost, earth abundance, availability of sodium source.^[^ ^1^ ^]^ However, conventional graphite anode with low theoretical specific capacity (<300 mAh g^−1^) can't meet the requirements of high energy and power density, limiting the large‐scale application of sodium batteries in energy storage field.^[^ ^2^ ^]^ The metallic sodium possessing a theoretical specific capacity (1166 mAh g^−1^) and low redox potential (–2.714 V vs standard hydrogen electrode), has attracted great interests as anode of sodium metallic batteries (SMBs). Whereas, several challenges remain to be addressed before their application of SMBs, such as the uncontrolled sodium dendrite growth, unrestrained volume expansion and unstable solid electrolyte interphase (SEI) layer owing to high activity of metallic sodium, which contributes to the inferior cycling stability and safety hazards.^[^ ^3^ ^]^

Various strategies have been proposed to handle aforementioned issues, including electrolyte optimization (high concentration electrolyte,^[^ ^4^ ^]^ electrolyte additives,^[^ ^5^ ^]^ solid state electrolyte^[^ ^6^ ^]^) and interface engineering (artificial SEI,^[^ ^7^ ^]^ alloy interface^[^ ^8^ ^]^) for stabilizing solid electrolyte interface, 3D scaffold engineering^[^ ^9^ ^]^ for accommodating the volume expansion. Although those methods alleviate corresponding problems through investigating formed Na deposition morphology at low areal capacity (<1 mAh cm^−2^) and areal current densities (<2 mA cm^−2^), they are rarely focusing on regulation of intrinsic factors responsible for the dendrite growth, including sodiophobic properties, highly inhomogeneous electric field distribution as well as nonuniform Na^+^ flux, especially under condition of high rate and high areal capacity. The sodiophilic materials, such as transmission metal of Au^[^ ^10^ ^]^ and carbon‐based materials with specific active group,^[^ ^11^ ^]^ are able to obtain the uniform plating through guiding initial nucleation behavior and growth during Na deposition. Whereas, these materials are just used as surface heterogeneous seeds to guide the Na deposition, the nonuniform Na^+^ flux and uncontrollable electric field remain to be addressed since they are crucial for Na planar growth. For example, metal tin is sodiophilic material, which can benefit Na deposition. However, some reported Na‐X alloy only acts as seeds for guiding Na ions deposition on the substrate^[^ ^8b^ ^]^ or as a protective layer,^[^ ^12^ ^]^ which is hard to obtain high deposition capacity for ultralong cycling life owing to repeated expansion and contraction of volume during Na plating and stripping.^[^ ^13^ ^]^ Therefore, achieving stable sodium metallic anode with high areal capacities at high current densities, is still a great challenge for high‐rate SMBs.

Herein, we report a metallic sodium foil (Na/NaSn) with 3D connected network integrating metallic sodium and Na_15_Sn_4_ alloy through an in situ alloy reaction. The 3D alloy network can not only provide an interpenetrated channel for fast sodium diffusion and act as stable skeleton for high areal capacity of Na deposition, but its porous structure is beneficial to accommodating the volume expansion and regulating Na^+^ flux during Na deposition. The alloy Na_15_Sn_4_ with ≈0.1 V potential difference can create a secondary electric field to drive the Na^+^ transfer, which benefits decreased ion concentration difference. In addition, the strong affinity between metallic Na and Na_15_Sn_4_ can help and favor the reduced Na deposition barrier, and the high sodium ion diffusion coefficient of Na_15_Sn_4_ enable fast kinetics for remarkable rate performances. The Na/NaSn anode can deliver an ultralong stripping/plating life for high deposition capacity (1600 h at 1 mA cm^−2^ for 4 mAh cm^−2^; 1600 h at 2 mA cm^−2^ for 2 mAh cm^−2^) with low deposition potential (24.5 mV) and fast kinetics (500 h at 4 mA cm^−2^ for 4 mAh cm^−2^). In addition, the Na/NaSn|NVP full cell can stably operate for 90 cycles with capacity retention of 95% at a current density of 1C and display superior rate performances. This work provides a promising potential for practical application of high rate SMBs with high capacity at high current densities.

## 2 Results and Discussion

**Figure 1:**
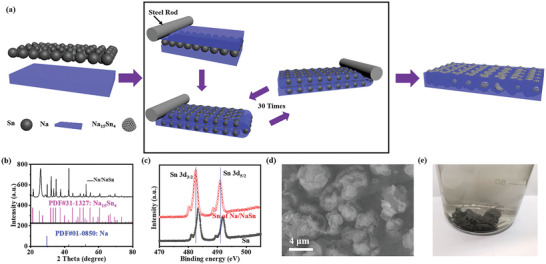
The synthesis process and physicochemical properties. a) The scheme of Na/NaSn material. b) The XRD patterns of Na/NaSn material. c) The XPS spectroscopy of Sn for Sn power and Na/NaSn, respectively. d) The SEM image of Na/NaSn. e) The optical image of Na/NaSn sheets after soaking in ethanol.

The Na/NaSn foil was prepared through repeated folding and calendaring of Sn powder (Figure S1, Supporting Information) and Na foil for 30 times, where the Na–Sn alloy can be simultaneously formed at room temperature under inert atmosphere, as illustrated in **Figure** **1**a. The metallic Na foil with bright silver luster immediately becomes greyish white after alloy reaction (Figure S2, Supporting Information). The atomic ratios of Na to Sn are set to be 30:4, 45:4, 90:4, which are based on the equation of (*x* + 15)Na + 4Sn = Na_(_ *_x_*_+15)_Sn_4_ demonstrated by X‐ray photoelectron spectroscopy （XRD） patterns (Figure 1b). The surplus Na is filled in the pores of alloy particles (Figure S3, Supporting Information) and interstice between interconnected Na_15_Sn_4_ particles. The X‐ray photoelectron spectroscopy (XPS) spectroscopy of pure Sn has two main peaks centered at 484.9 and 493.3 eV for Sn3d_5/2_ and Sn3d_3/2_,^[^ ^14^ ^]^ respectively, and then shifted to lower positions after alloyed with Na, indicative of the formation of Na_15_Sn_4_ alloy (Figure 1c). The morphologies of metallic Na foil and Na/NaSn foil were observed by scanning electron microscope (SEM, Figure 1d). Compared to morphology of metallic Na (Figure S4, Supporting Information), the Na/NaSn foil exhibits more rough surface with some exposed micro‐size Na_15_Sn_4_ alloy particles demonstrated by energy dispersive X‐ray spectroscopy (EDX) mappings (Figure S5, Supporting Information). The morphology of dealloyed Sn particles (Figure S6a, Supporting Information) shows interconnected network with specific surface area of 10 m^2^ g^−1^ (Figure S6b, Supporting Information), and the Sn particles can be closely connected to form sheets (Figure 1e). This special interconnected skeleton can act as stable intrinsic host for uniform Na nucleation and optimize the electric field distribution owing to the potential difference (0.1 V)^[^ ^15^ ^]^ between Na_15_Sn_4_ and Na, which contributes to reduced Na dendrite and alleviation of volume expansion during Na deposition. The Na/NaSn foil discussed as followed mainly refers to the one with mass ratio of 45:4 (Na:Sn) owing to its good electrochemical performances (Figure S7, Supporting Information). Since the thickness of Na/NaSn electrode is set as 100 µm, the calculated specific capacity is about 11 mAh g^−1^.

**Figure 2:**
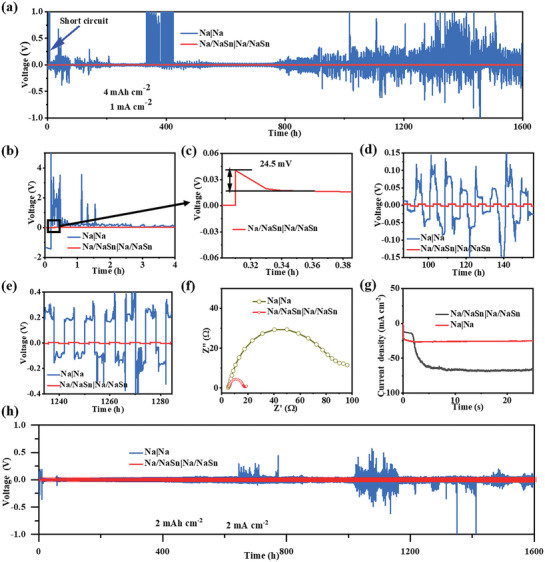
Electrochemical characterization of the electrodes for Na stripping/plating: a) The stripping/plating curves for Na|Na and Na/NaSn|Na/NaSn symmetrical batteries. b) The galvanostatic voltage profiles of Na and Na/NaSn electrode and its enlarged voltage profile. c) at initial stripping stage. d,e) The galvanostatic voltage profiles of Na and Na/NaSn electrode ranging from 100 to 140 h and from 1240 to 1280 h under 4 mAh cm^−2^ at 1 mA cm^−2^, respectively. f,g) The EIS measurements and CA measurements for Na|Na and Na/NaSn|Na/NaSn symmetrical cells, respectively. h) The stripping/plating curves for Na|Na and Na/NaSn|Na/NaSn symmetrical batteries under 2 mAh cm^−2^ at 2 mA cm^−2^.

To evaluate the structural advantages for metallic sodium deposition, the stripping and plating measurements for Na|Na and Na/NaSn|Na/NaSn symmetrical batteries were carried out. **Figure** **2**a shows the charge/discharge voltage profiles for Na/NaSn and metallic Na electrode with a high areal capacity of 4 mAh cm^−2^ (≈34 µm thickness) at a current density of 1 mA cm^−2^. The Na/NaSn|Na/NaSn symmetrical battery can keep ultralong cycling stability for 1600 h without potential drift, while the metallic Na|Na battery appears soft short circuit at first stripping/plating circles, indicating that the framework of Na–Sn alloy is favorable for stable Na deposition. Meanwhile, it can be seen that lower voltage hysteresis of 24.5 mV at initial charge stage is observed for Na/NaSn|Na/NaSn battery, but the Na|Na battery shows a near 5 V voltage hysteresis (Figure 2b,c), implying that the Na–Sn alloy skeleton is qualified with excellent sodiophilic property, which is beneficial to lowering nucleation barrier. Figure 2d,e displays the corresponding enlarged voltage profiles ranging from 100 to 140 h and from 1240 to 1280 h for Na/NaSn|Na/NaSn and Na|Na batteries, respectively. The Na/NaSn|Na/NaSn batteries has lower stripping/plating potentials (8 mV at 100 h and 9 mV at 1240 h, Figures S8a,b, Supporting Information) and more flat stripping/plating curves compared to those of the Na|Na batteries (Figure S8c,d, Supporting Information), suggesting the homogeneous and stable Na deposition on surface Na–Sn alloy framework. The electrochemical impedance spectroscopy (EIS) displays that the Na/NaSn|Na/NaSn battery has a smaller diameter of half cycle than that of Na|Na battery, suggesting the improved charge transfer capability owing to the excellent sodiophilic property of Na–Sn alloy (Figure 2f).^[^ ^16^ ^]^ After 50 cycles, the diameter for Na/NaSn|Na/NaSn battery decreases, indicative of the reduced charge resistance, further demonstrating the outperformed cycling stability and low stripping/plating potential (Figure S9, Supporting Information). The early stage of Na nucleation and growing process on the surface of electrodes were investigated by chronoamperometry method, as shown in Figure 2g. The Time–current curve derived at an overpotential of 0.2 V for Na/NaSn|Na/NaSn battery shows higher current density (68 mA cm^−2^) than that (26 mA cm^−2^) of Na|Na battery, which indicates that Na–Sn alloy framework exhibits excellent ion conductivity for good rate performances.^[^ ^17^ ^]^ To further evaluate the structural advantages for fast electrochemical kinetics, the measurement of higher areal current density was carried out for Na|Na and Na/NaSn|Na/NaSn symmetrical batteries, as shown in Figure 2h. Apparently, compared to the unstable stripping/plating process of Na|Na battery, the Na/NaSn|Na/NaSn battery can stably operate for 1600 h without obvious voltage drift at 2 mA cm^−2^ for 2 mAh cm^−2^, demonstrating its remarkable Na‐ion transfer kinetics.

**Figure 3:**
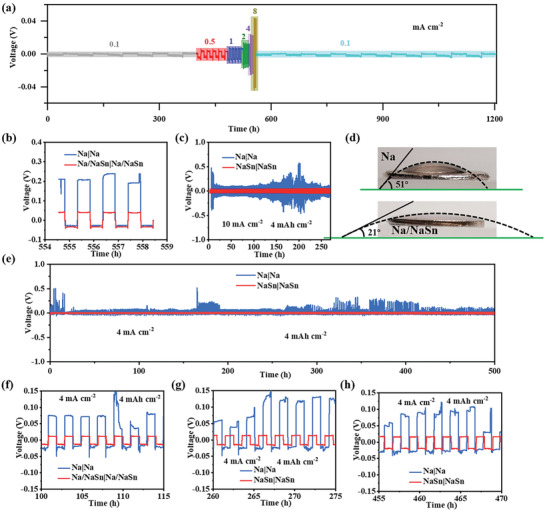
The electrochemical kinetics and long cycling stability. a) The rate performances for Na|Na and Na/NaSn|Na/NaSn symmetrical batteries under 4 mAh cm^−2^. b) The enlarged galvanostatic voltage profiles of Na|Na and Na/NaSn|Na/NaSn symmetrical batteries ranging from 555 to 558 h under 4 mAh cm^−2^ at 10 mA cm^−2^. c) The stripping/plating curves for Na|Na and Na/NaSn|Na/NaSn symmetrical batteries under 4 mAh cm^−2^ at 10 mA cm^−2^. d) The contact angle measurements for Na and Na/NaSn electrode. e) The stripping/plating curves for Na|Na and Na/NaSn|Na/NaSn symmetrical batteries under 4 mAh cm^−2^ at 4 mA cm^−2^ and f–h) their corresponding galvanostatic voltage curves at different cycling states, respectively.

The **Figure** **3**a shows the rate performance of Na/NaSn|Na/NaSn symmetrical batteries at different current densities ranging from the 0.1 to 8 mA cm^−2^ under a fixed capacity of 4 mAh cm^−2^. It is notable that Na/NaSn|Na/NaSn battery reveals superior rate performances and more stable voltage profile with smaller voltage hysteresis compared to that of Na|Na (Figure S10, Supporting information). Even at striping/plating at 8 mA cm^−2^, the deposition voltage is only 50 mV (Figure 3b) and then turns back to the pristine state when the current density becomes 0.1 mA cm^−2^, indicating Na/NaSn anode exhibits remarkable electron/ion transfer properties, which are responsible for stable Na deposition at high areal current densities. When tested at ultrahigh areal density of 10 mA cm^–2^, Na/NaSn| Na/NaSn battery can cycle for 250 h with low potential of 50 mV (Figure 3c), further confirming the fast kinetics and good rate performances. The wettability of electrolyte on the surface of Na/NaSn and Na electrode is characterized by contact angles (Figure 3d).^[^ ^18^ ^]^ Notably, the contact angle (21°) of Na/NaSn is smaller than that (51°) of Na electrode, further implying lower nucleation barrier, which contributes to the excellent rate performances. The long stripping/plating measurements for both electrodes are investigated at 4 mA cm^−2^ for 4 mAh cm^−2^, as shown in Figure 3e. The Na/NaSn| Na/NaSn battery can stably cycle for 500 h with no obvious voltage fluctuation, and the stripping voltages are 11, 12, 14 mV at different time ranging from 100 to 150 h, 260 to 275 h and 455 to 470 h, respectively, while the metallic Na|Na symmetrical battery delivers a high overpotential and turbulent stripping/plating curves, which stems from high interfacial resistance and uncontrolled Na dendrite (Figure 3f–h). In order to explore the practical application of electrode, the electrochemical performance of Na/NaSn symmetrical battery with 50 µm thickness was explored, which can stably cycle for 500 h without obvious potential drift (Figure S11, Supporting Information).

Such unique structural design allows fast sodium‐ion diffusion over the entire electrode and enables stable sodium stripping/plating cycling at high current densities without sodium dendrite growth and high electrochemical deposition capacities due to its multiple advantages: First, the strong sodiophilic interphase is beneficial to decreasing nucleation barrier and lowering electrochemical potential, while the interpenetrated network can provide effective ion transfer pathway over the entire electrode, and rich pores accommodate high‐capacity metallic Na deposition and alleviate the volume expansion. Second, the potential difference between the Na and Na_15_Sn_4_ can offer additional driving force for Na ion transfer along the pathway.^[^ ^15^, ^19^ ^]^ Third, the alloy Na_15_Sn_4_ is less reactive with the electrolyte due to its higher electrochemical potential than metallic sodium (Figure S12, Supporting Information),^[^ ^20^ ^]^ which displays ultralong cycling life owing to less electrolyte consumption.

**Figure 4:**
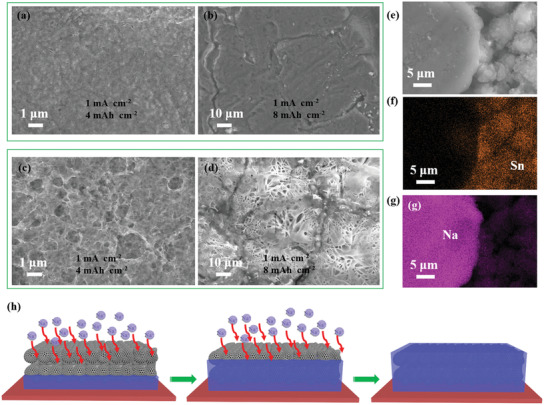
The exploration of mechanism of Na deposition on the Na_15_Sn_4_ electrode. a,c) The SEM images for Na and Na/NaSn electrode with deposition of 4 mAh cm^−2^ and b,d) 8 mAh cm^−2^ after an initially stripped capacity of 2 mAh cm^−2^. e) The SEM image of Na/NaSn electrode after removal of part deposited Na and corresponding EDX mappings of Sn f) and Na g), respectively. h) The mechanism of Na deposition on Na/NaSn alloy framework.

In order to explore the morphological evolution of Na deposition on Na and Na/NaSn electrode (The electrodes have been firstly stripped with a capacity of 2 mAh cm^−2^), the ex situ SEM was carried out (**Figure** **4**). After a capacity of 4 mAh cm^−2^ of Na deposition, the Na/NaSn electrode exhibits a smooth surface without obvious Na dendrite, while Na electrode shows porous structure (Figure 4a,c). When further depositing with additional 4 mAh cm^−2^ on the Na/NaSn electrode, the planar surface is still preserved (Figure 4b). In contrast, the Na dendrite protrusions with messy morphologies covered on surface were observed for metallic Na electrode (Figure 4d), which indicates that Na–Sn alloy skeleton can not only favor uniform Na nucleation and Na growth, but accommodate ultrahigh deposition capacities. Figure 4e–g give the EDX mappings of Na and Sn elements for Na/NaSn electrode with a deposition capacity of 8 mAh cm^−2^. Obviously, the Na–Sn alloy particles gather closely and ultrahigh‐capacity Na can uniformly deposit on the Na–Sn alloy.

To demonstrate the structural merits during cycling process, the Na|Na and Na/NaSn|Na/NaSn batteries were stripped/plated for ten cycles at different current densities (1, 4, 8 mA cm^−2^) at a capacity of 4 mAh cm^−2^ and their corresponding depositing appearances were further observed. The Na/NaSn electrode remains planar surface at 1 and 4 mA cm^−2^ (Figure S13a,b, Supporting Information), explaining its superior rate performances and cycling stability, while the metallic Na electrode shows Na dendrite and porous structure, which would contribute to dead Na when operated for long stripping/plating cycles (Figure S13d,e, Supporting Information). Even measured at 8 mA cm^−2^, the surface of Na/NaSn electrode covered by intimately connected small particles is still flat, while the Na electrode displays serious Na protrusions and aggravated porous structure (Figure S13c,f, Supporting Information). These phenomena imply that Na–Sn alloy can act as favorable framework for stable and high‐capacity Na deposition at high current densities. Therefore, the mechanism of Na deposition on the Na–Sn alloy can be included that (Figure 4h): sodium ions firstly adsorb on surface of Na_15_Sn_4_ through solid‐liquid interface, then penetrate entire Na–Sn alloy network into Na substrate, and finally form Na planar. In order to study favorable framework, XPS measurements are carried out at different depths, such as 0, 10, 20, and 30 nm. The full spectra (Figure S14, Supporting Information) mainly contain five elements of Na, P, C, O, F. The fitted spectra of element F show two peaks assigned to Na—F bond and P—F bond, and the P—F bond is related to remained electrolyte (NaPF_6_). As an increase of etching depth, the intensity of Na—F bond of Na/NaSn electrode becomes weaker compared to those of Na electrode, indicating that Na/NaSn electrode has more thinner SEI film (Figure S15a, Supporting Information).^[^ ^21^ ^]^ From the fitted curves of Na (Figure S15b, Supporting Information), peak intensity of Na—F bond for Na/NaSn electrode is similar at different testing depth, which is far lower than those of pure Na electrode at 0, 10, 20 nm. This result further demonstrates the lower activity of Na–Sn alloy compared with pure Na metal. In the XPS spectra of P (Figure S15c, Supporting Information), there are two peaks assigned to —PF_6_ and O—P═O,^[^ ^5^ ^]^ and the total contents of P are 2.4% for Na electrode and 0.8% for Na/NaSn electrode, respectively. As for element O (Figure S16a, Supporting Information), the spectra of Na anode can be deconvoluted into four peaks of C—C—O, C—O—Na, Na_2_O, Na kll, while Na/NaSn anode exhibits less functional groups, which could be attributed to the different reactivity. From the fitted curves of element C (Figure S16b, Supporting Information), it can be deconvoluted into four peaks of —C—C, C—O, Na_2_CO_3_, C—F.^[^ ^22^ ^]^ It is notably that Na/NaSn electrode exhibits less peaks and weaker peak intensity than those of Na electrode, indicating that the formation of alloy can weaken the reaction between electrode and electrolyte.

**Figure 5:**
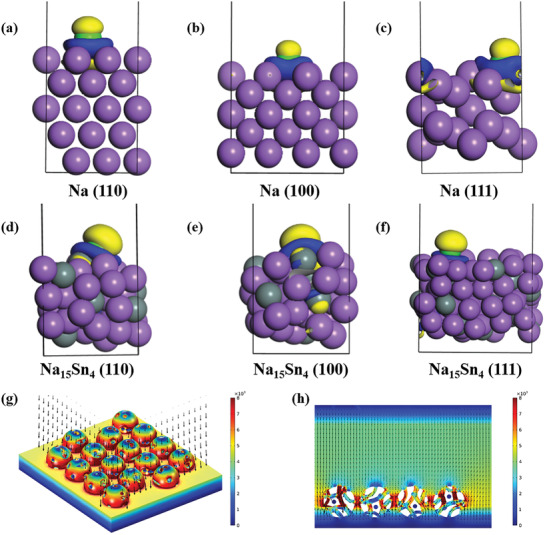
The theoretical calculation and numerical simulation for exploring uniform Na deposition. a–f) The differential charge density of a) Na(110), b) Na(100), c) Na(111), d) Na_15_Sn_4_(110), e) Na_15_Sn_4_(100), f) Na_15_Sn_4_(111). g) The exterior and section view h) of numerical simulation of Na^+^ flux.

To further explore the intrinsic reasons of the Na/NaSn anode with excellent electrochemical performances, the first principle calculation (DFT) was carried out.^[^ ^23^ ^]^ The adsorption properties of single Na on metallic Na and Na_15_Sn_4_ alloy surfaces have been investigated, where the calculated crystal faces of Na and Na_15_Sn_4_ are 100, 111, 110 (Figure S17, Supporting Information). The calculated binding energy *E*_b_ is 0.58 eV on Na (100), 0.46 eV on Na (111), and 0.35 eV on Na (110), while the calculated *E*_b_ for Na_15_Sn_4_ is 1.45 eV on (110), 1.17 eV on (100), and 0.19 eV on (111). Clearly, Na_15_Sn_4_ alloy can remarkably strengthen Na adsorption since both (110) and (100) can offer binding capability as strong as that in bulk sodium, which indicates that (110) and (100) of Na_15_Sn_4_ is preferred crystal plane for uniform Na deposition, demonstrated by XRD patterns of depositing metallic Na (Figure S18, Supporting Information). The images of differential charge density (**Figure** **5**a–f) for different crystal faces (110, 100, 111) of Na_15_Sn_4_ show that more electrons accumulate around Sn compared to Na, which is confirmed by calculated Mulliken charge (Table S1, Supporting Information), indicating that Na^+^ can be intimately adsorbed by Na–Sn bonds. We also noticed that Na favors the bonding with Sn on the surface, especially at active sites when Sn is presented at the surface and subsurface as in (110) and (100).

Numerical simulations of the Na^+^ flux distribution for Na_15_Sn_4_ electrode were carried out to understand deposition behavior. Notably, Na ion flux is inclined to gather the surface of Na_15_Sn_4_ (Figure 5g), indicating that Na_15_Sn_4_ alloy has strong affinity with Na^+^. In addition, Figure 5h shows the Na^+^ flux in pylome is more homogeneous and enhanced, suggesting that porous architecture with additional electronic field can improve the magnitude of Na^+^ flux for planar Na deposition. When increasing plating capacity, deposited Na can still form planar surface, and the adjacent area between Na_15_Sn_4_ particles exhibits more homogeneous electric field (Figure S19, Supporting Information), indicating interconnected alloy network can optimize electric field distribution for uniform Na deposition.

**Figure 6:**
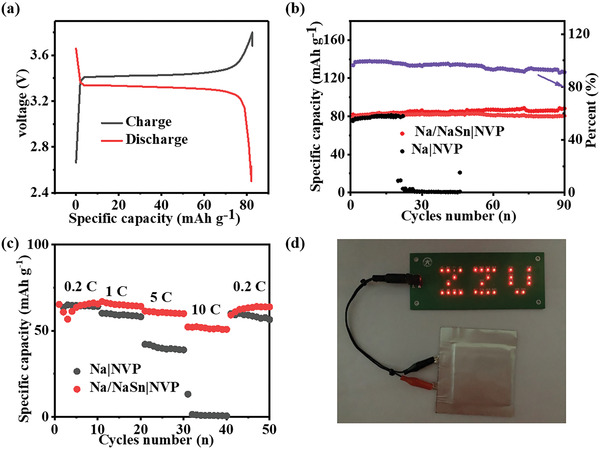
The practical application of Na/NaSn electrode. a–c) The charge–discharge curves, cycling performance at 1C and rate performance of Na/NaSn|NVP and Na|NVP full cell, respectively. d) The LED subtitles were lighted by soft‐package batteries Na/NaSn|NVP.

To investigate the practical application of Na/NaSn electrode, the full batteries were assembled with NVP (Na_3_V_2_(PO_4_)_3_) as cathode (Figure S20, Supporting Information). The full battery Na/NaSn|NVP was operated at 1C (≈2 mA cm^−2^) in the potential window of 2.5–3.8 V (**Figure** **6**a) and the active NVP loading is 11 mg cm^−2^ (≈0.9 mAh cm^−2^). The full cell can stably charge and discharge for 90 cycles and deliver a capacity of 80 mAh cm^−2^ (Figure 6b). The rate performances for Na/NaSn and Na were carried out at 0.2C, 1C, 5C, 10C, 0.2C, respectively. It is notable that the Na/NaSn|NVP full cell reveals an excellent rate performance (Figure 6c), indicating the favorable Na_15_Sn_4_ alloy skeleton. The soft package of Na/NaSn|NVP was prepared in a size of 6 × 6 cm. This full cell can light a LED panel (Figure 6d), which further confirms its practical properties.

## 3 Conclusions

In summary, a special metallic Na anode (Na/NaSn) integrating 3D connected Na–Sn alloy network and metallic Na was facilely designed through spontaneous reaction of Na and Sn at room atmosphere, enabling stable stripping/plating measurements for long life at high areal capacity and high current densities. Owing to the good Na affinity, Na_15_Sn_4_ can decrease the Na nucleation barrier for lower deposition potential, while the auxiliary potential difference between Na_15_Sn_4_ and Na can create an additional driving force to regulate the Na‐ion flux for uniform Na deposition. The 3D Na–Sn alloy network with high Na‐ion diffusion coefficient can provide a penetrated pathway for fast Na diffusion over the entire framework, and its porous structure can accommodate the volume expansion during Na deposition. Through regulation of ion flux, electric field and sodiophilic property, the Na/NaSn anode with free dendrite can be realized at a high areal capacity of 4 mAh cm^−2^ for ultralong stripping/plating cycles and enable fast kinetics. The Na/NaSn|NVP full cell can present a remarkable cycling stability and an excellent rate performance. This work provides a novel strategy to facilely design metallic Na anode for high areal capacities and excellent rate performances, giving the potential application of energy storage.

## Conflict of Interest

The authors declare no conflict of interest.

## Supporting information

Supporting Information

## Data Availability

The data that support the findings of this study are available in the supplementary material of this article.
