# Supplementary material for: Synergistic Manipulation of Na+ Flux and Surface‐Preferred Effect Enabling High‐Areal‐Capacity and Dendrite‐Free Sodium Metal Battery
Source: Adv Sci (Weinh). 2022 Jan 9;9(7):2103845. doi: 10.1002/advs.202103845 (PMC8895136; doi:10.1002/advs.202103845)
Supplement: Supplementary file 1 — Supporting Information [file ADVS-9-2103845-s001.pdf]

© 2020 Wiley-VCH GmbH

ADVANCED SCIENCE
Open Access

Supporting Information

for *Adv. Sci.,* DOI: 10.1002/advs.202103845

Synergistic Manipulation of Na^+ Flux and Surface-Preferred Effect Enabling High-Areal-Capacity and Dendrite-Free Sodium Metal Battery

Qianzheng Jin, Hongfei, Lu Zhili Zhang, Jing Xu, Bing Sun, Yang Jin* and Kai Jiang**

Supporting Information

Synergistic Manipulation of Na^+ Flux and Surface-Preferred Effect Enabling High-Areal-Capacity and Dendrite-Free Sodium Metal Battery

Qianzheng Jin, Hongfei, Lu Zhili Zhang, Jing Xu, Bing Sun, Yang Jin* and Kai Jiang**

Experimental Section

Materials Synthesis: The Na/NaSn foil was realized via repeated folding and calendaring method, where the Sn power was firstly spread on the Na foil and then rolled repeatedly for 30 times with 100 μm thickness. The molar ratios of Na to Sn were set as 30:4, 45:4, 90:4, which principle was based on the reaction equation of (15+x)Na + 4Sn =$Na_{(15+x)}Sn_4$, and the corresponding products can be assigned as Na/NaSn-1, Na/NaSn, Na/NaSn-2. As a comparison, the Na foil was rolled with 100 μm thickness. The cathode NVP ($Na_3V_2(PO_4)_3$) was prepared according to the previous paper.[1]

Materials Characterization: The physical morphologies and microstructures of materials were tested by scanning electron microscope (SEM) and transmission electron microscope (TEM) equipped with energy-dispersive spectrometer (EDS). The crystal structures were observed by X-ray diffraction (XRD) equipped with a radiation (λ= 1.5406 Å) over the 2 range of 10-60°. The elemental composites and chemical states were characterized using the X-ray photoelectron spectroscopy (XPS). The specific surface area of porous materials was measured by BET analysis.

Electrochemical Tests: The 2032-type cells were assembled in glovebox under Ar atmosphere. The electrolyte (40 μL) was 1 mol L^{-1} $NaPF_6$ in 1,2-Dimethoxyethane (DME) and separator was celgard 2400. The cathode electrodes were prepared using NVP, super P, binder (PVDF) at ratio of 8:1:1 and corresponding loading was about 11 mg cm^{-2}. The full cells were assembled with NVP as cathode, Na foil or Na/NaSn foil as anode and celgard 2400 as separator, which were performed at different current densities. The galvanostatic charge/discharge performances were carried out on LANHE CT2001A battery testing system. The electrochemical dynamics was characterized by electrochemical impedance spectroscopy (EIS) at range of 0.1 Hz - 1 MHz.

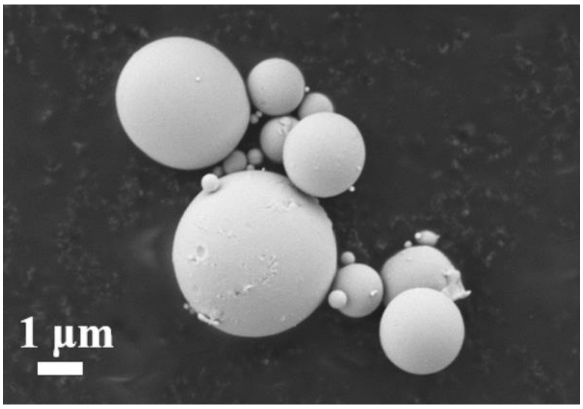

Figure S1. The SEM image of Sn power.

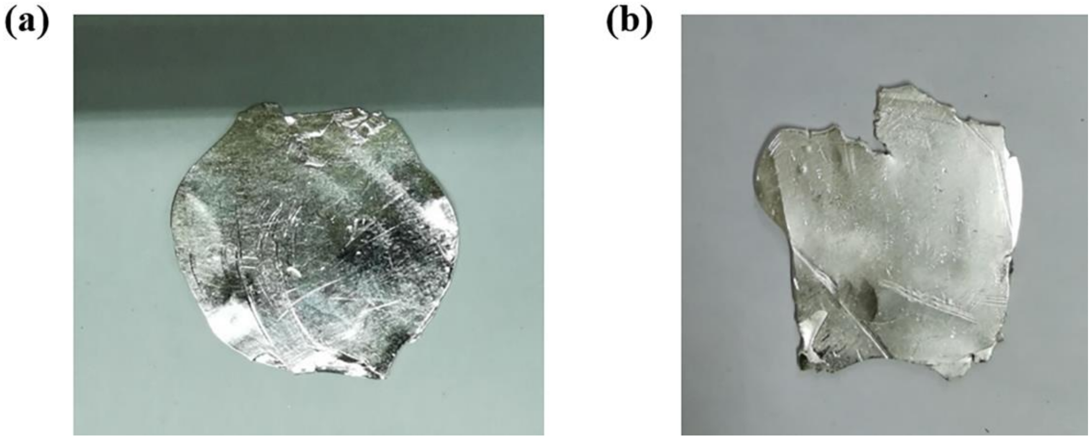

Figure S2. a) The optic graphs of Na foil and Na/NaSn foil (b).

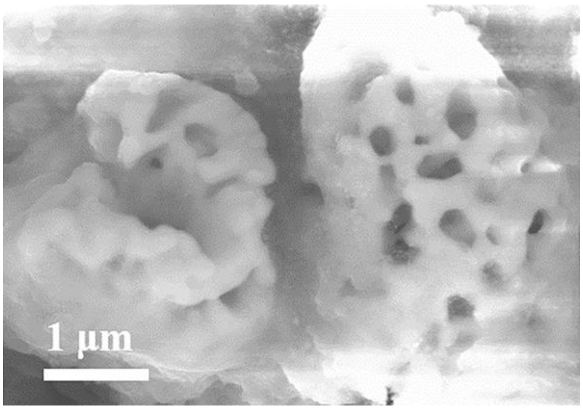

Figure S3. The SEM image of Na/NaSn after stripping Na.

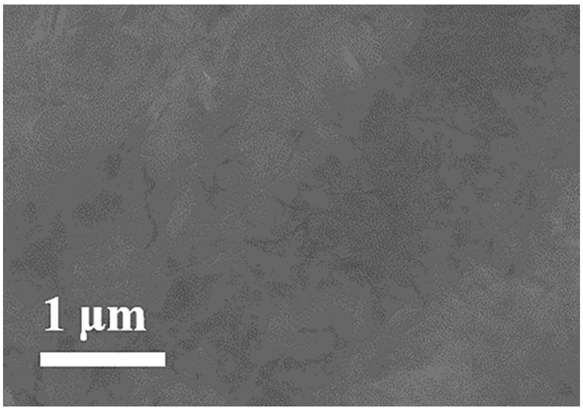

Figure S4. The SEM images of metallic Na.

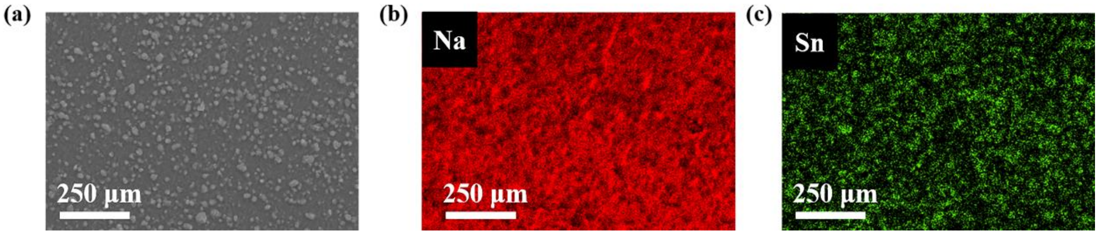

Figure S5. The SEM image of Na/NaSn (a) and corresponding mappings of Na and Sn (b, c).

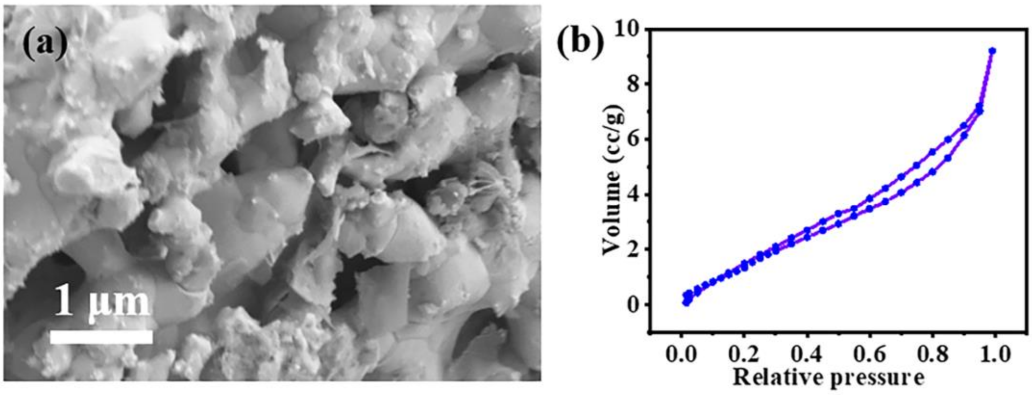

Figure S6. a, b) The SEM image and BET curve of dealloyed Sn particles after soaking in ethanol.

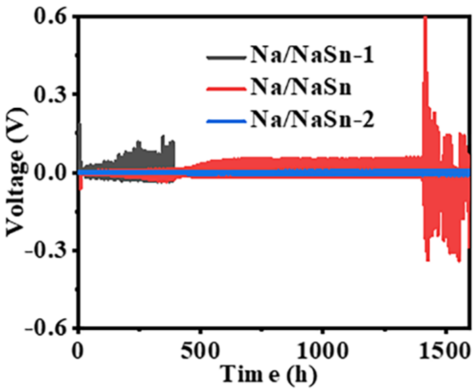

Figure S7. The voltage profiles of Na/NaSn-1, Na/NaSn, Na/NaSn-2.

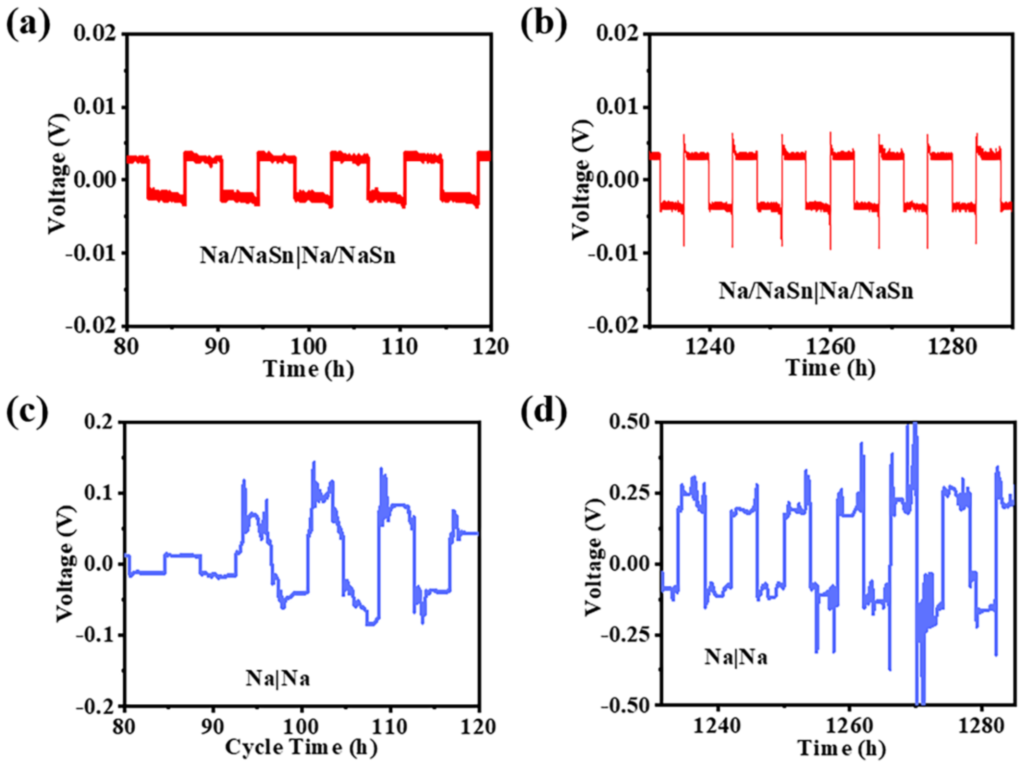

Figure S8. a, c) The corresponding enlarged voltage profiles ranging from 100 to 140 h and (b, d) from 1240 to 1280 h for Na/NaSn|Na/NaSn and Na|Na batteries, respectively.

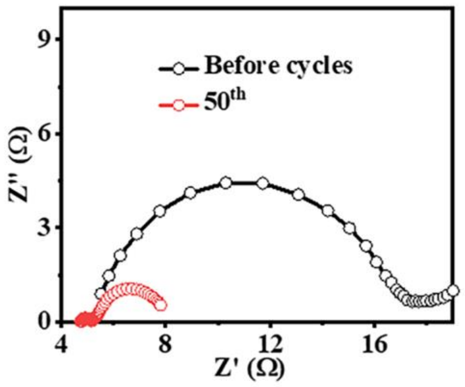

Figure S9. The EIS measurements of Na/NaSn|Na/NaSn battery before cycles and after 50 cycles.

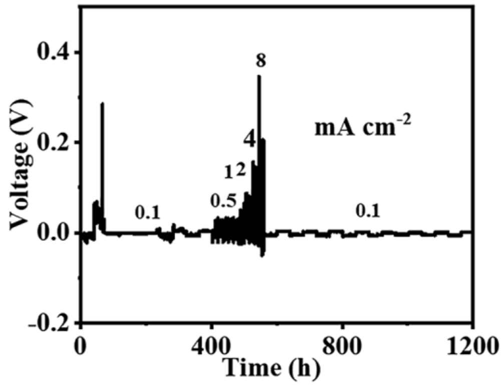

Figure S10. The rate performance of Na |Na battery.

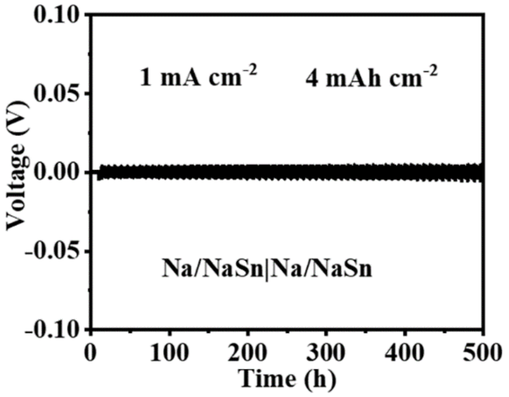

Figure S11. The cycling performances of Na/NaSn| Na/NaSn symmetrical battery with a thickness of 50 μm.

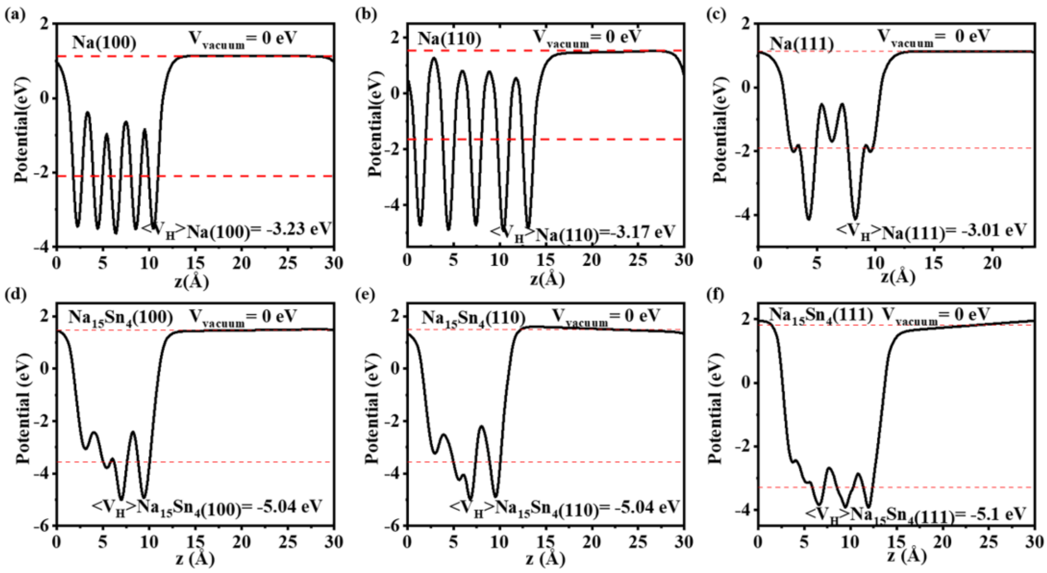

Figure S12. The average electrostatic potentials of Na and $Na_{15}Sn_4$ versus the vacuum energy level at (100), (110), (111).

As the energy difference between the Fermi energy level of the anode and the lowest unoccupied molecular orbital (LUMO) of the electrolyte solution is the driving force behind the formation of the SEI film,[2] the higher Fermi energy level of anode means the higher tendency to form the SEI film. The Fermi energy level of the anode can be illustrated by the work function of anode, which is the minus of the average electrostatic potential of anode versus the vacuum energy level ($E_{wf} = V_{vacuum} - \langle V_H \rangle_{anode}$). The work function of Na and $Na_{15}Sn_4$ surface can be obtained from the average electrostatic potential shown in Figure S12. From Figure S12, we can see that the work function of Na(100), Na(110), Na(111) and $Na_{15}Sn_4$(100), $Na_{15}Sn_4$(110), $Na_{15}Sn_4$(111) surface are 3.23 eV, 3.17 eV, 3.01 eV and 5.04 eV, 5.04 eV, 5.10 eV, respectively. The work function of Na surfaces is obviously smaller than the $Na_{15}Sn_4$ surfaces, indicating that the higher Fermi energy level of Na surfaces. The higher Fermi energy level of Na surfaces provides the larger driving force to form the SEI film.

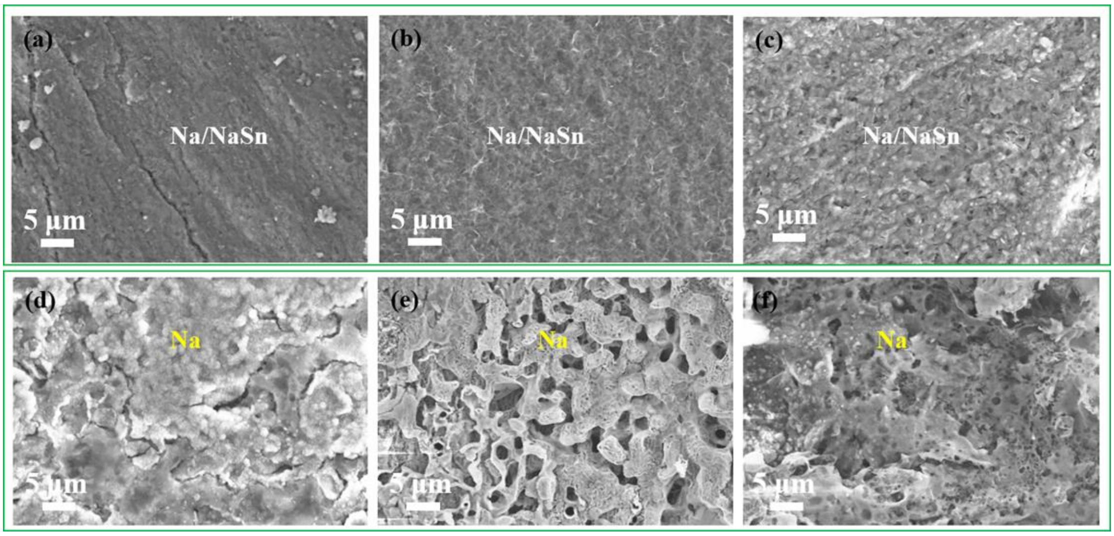

Figure S13. The SEM images of Na/NaSn and Na electrode after 10 cycles at 1 mA cm^{-2} (a, d), 4 mA cm^{-2} (b, e), 8 mA cm^{-2} (c, f), respectively.

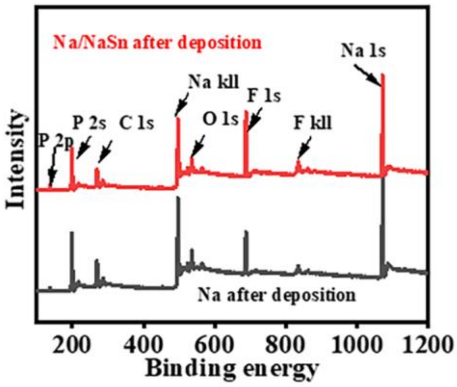

Figure S14. The full XPS spectra of Na foil and Na/NaSn foil after deposition of Na

for 4 mAh cm^{-2}.

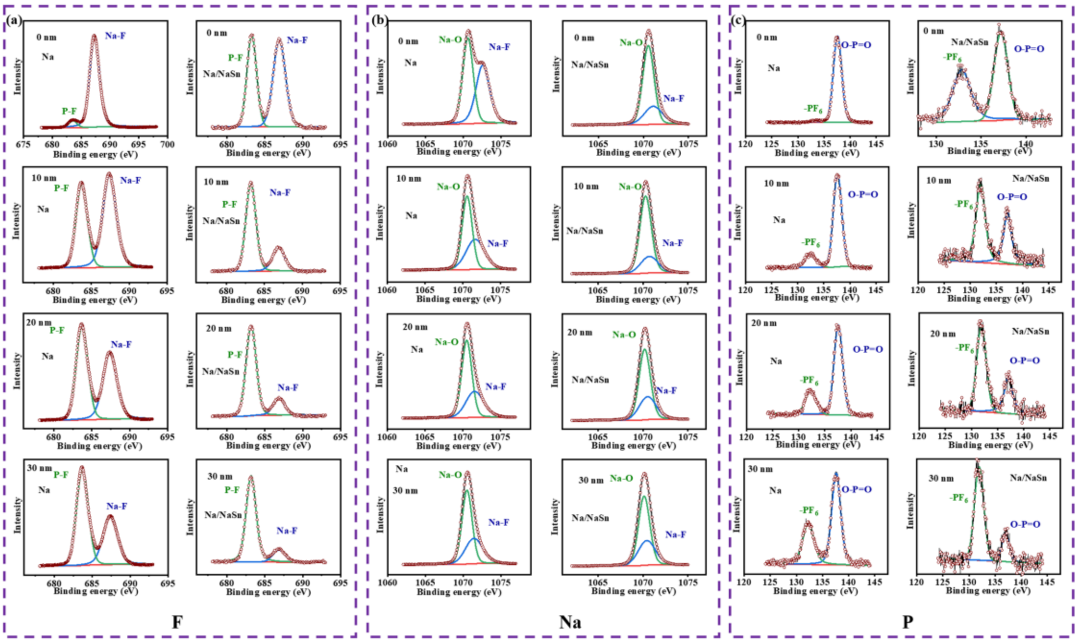

Figure S15. a, b, c) The fitted XPS spectra of elements F, Na, P for Na anode and Na/NaSn anode at different etching depths of 0 nm, 10 nm, 20 nm, 30 nm.

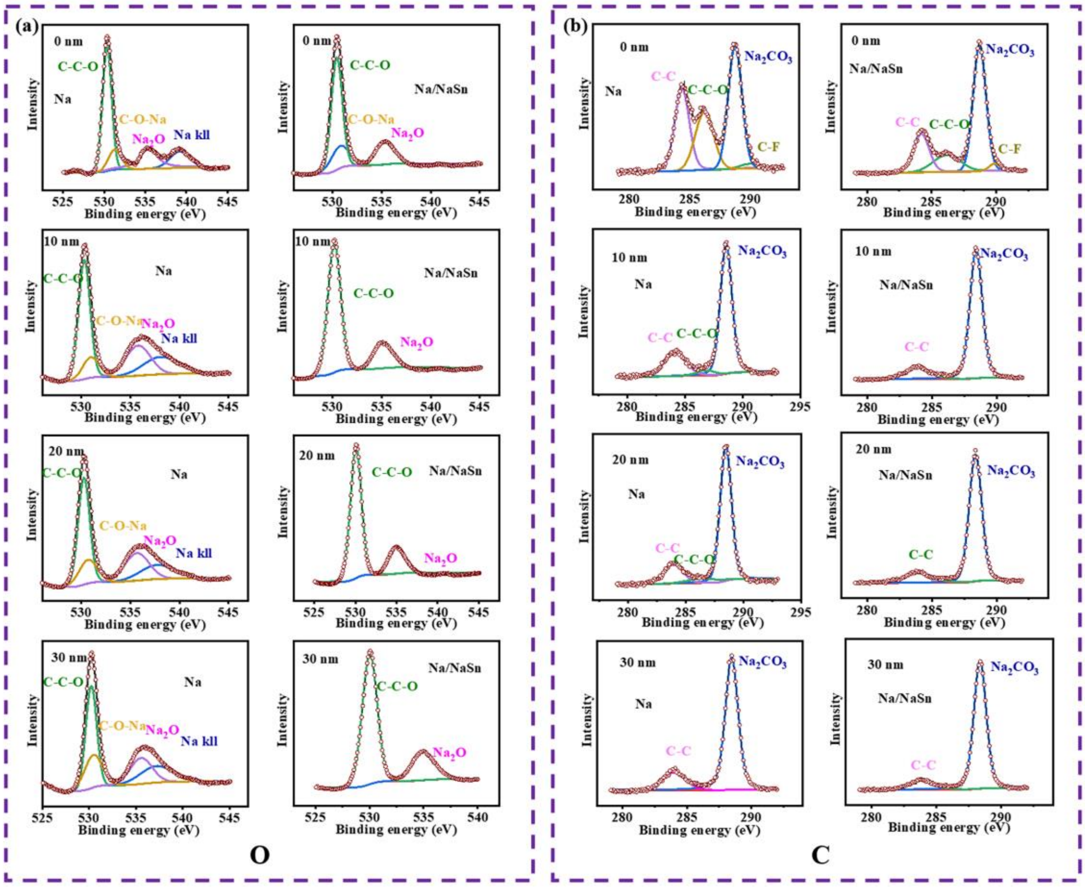

Figure S16. a, b) The fitted XPS spectra of elements O and C for Na anode and Na/NaSn anode at different etching depths of 0 nm, 10 nm, 20 nm, 30 nm.

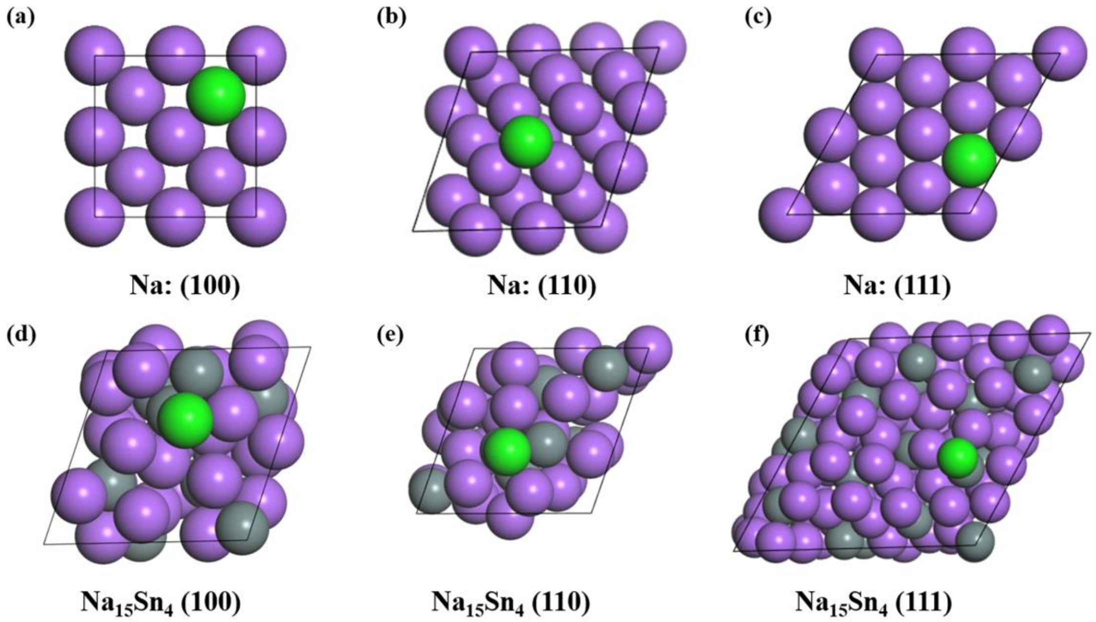

Figure S17. Optimized geometries for single Na adsorbed on (a) Na(100), (b) Na(110), (c) Na(111), (d) $Na_{15}Sn_4$(100), (e) $Na_{15}Sn_4$(110), (f) $Na_{15}Sn_4$(111). Na and Sn are shown as purple and grey balls, and the adsorbed Na as green.

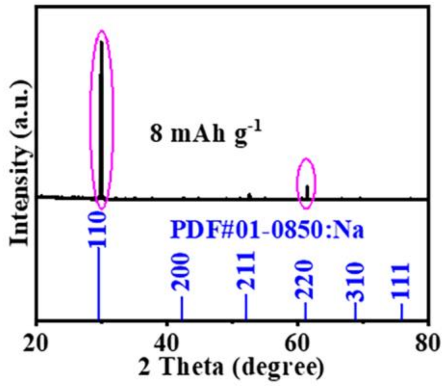

Figure S18. The XRD pattern of Na deposition on Na/NaSn with 8 mAh g^{-1}.

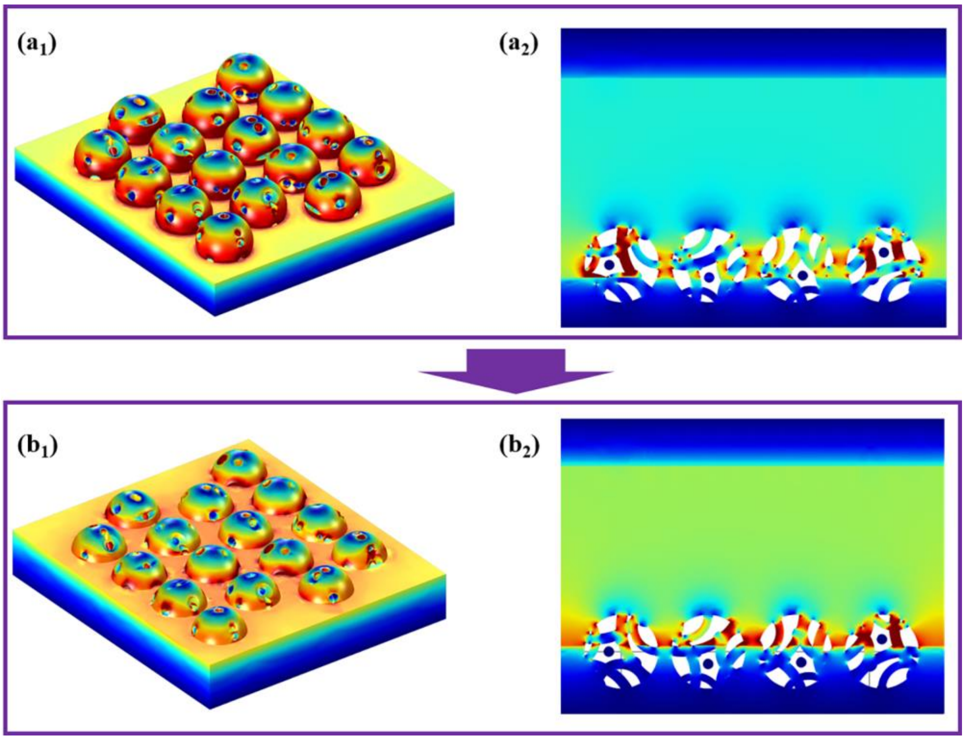

Figure S19. a_1, a_2) The exterior and section view of numerical simulation of Na^+ flux. b_1, b_2) The exterior and section view of numerical simulation of Na^+ flux after Na deposition.

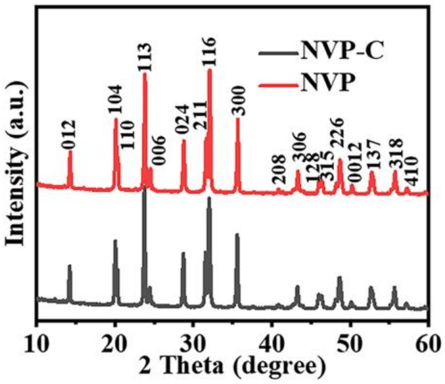

Figure S20. The XRD patterns of NVP and commercial NVP-C.

Table S1. The calculated Mulliken charge in unit of e.

surfaces	Adsorbed Na	1st Nearest	2nd Nearest
Na(110)	+0.10	Na:-0.16	Na:-0.10
Na(111)	+0.02	Na:-0.12	Na:+0.05
Na(110)	-0.12	Na:-0.07	Na:+0.06
$Na_{15}Sn_4$(100)	+0.33	Sn:-0.66	Na:+0.27
$Na_{15}Sn_4$(110)	+0.27	Sn:-0.66	Na:+0.19
$Na_{15}Sn_4$(111)	+0.27	Sn:-0.77	Na:+0.24

Computational Methods:

The first-principles calculations based on the Density Functional Theory (DFT) were performed using the CASTEP.[3] The Perdew-Burke-Ernzerhof (PBE) exchange-correlation functional[4] within the generalized gradient approximation (GGA) was employed to describe the exchange-correlation energy. The projector-augmented-wave (PAW)[5] method was adopted for the pseudopotentials. The energy cutoff for the plane wave basis expansion was set to 450 eV. The convergence criteria for total energy and force on each atom were set as 10^{-4} eV and 0.02 eV/Å, respectively. Slab model was constructed with a vacuum layer of 15 Å in the z direction to avoid the interaction between layers. The sampling in the Brillouin zone for all surface slabs was set with 3×3×1 by the Monkhorst-Pack method, but it is extended to 4×4×4 for bulk sodium. The van der Waals interaction has been considered using the Grimme dispersion scheme. To evaluate the computational setting, the binding energy for body centre sodium was calculated to be 1.22 eV, which is close to experimental data (1.13 eV).

Na surfaces have been investigated with Na(110), Na(111) and Na(100), as modelled by (2×2×1) supercells with a typical thickness of 9-12 Å, whose surface dimension is around 11.15 Å×11.15 Å, 12.14 Å×12.14 Å and 12.87 Å×12.87 Å for (110), (111) and (100). Similarly, $Na_{15}Sn_4$ was studied using low-indexed surfaces (100), (110) and (111), which were modelled by supercells with similar size and thickness as Na surfaces. In all cases, Na adsorption was studied using single Na adsorbed over these surfaces, followed by geometry optimization and electronic calculations under the same convergence criteria.

Before discussing the adsorption, we calculated binding energy E_b for bulk sodium (a=4.29 Å), with E_b=1.22 eV, which is consistent with experimental data (1.13 eV), but slightly larger than early reports (1.08 eV). Based on optimized geometries, the calculated adsorption energy was defined as $E_{ads}=E_{Na\text{-}Surface}-E_{Surface}-E_{Na}$, where $E_{Na\text{-}Surface}$, $E_{Surface}$ and E_{Na} are calculated total energies for Na adsorbed on the surface, clean surface and single Na derived from bulk body-centre sodium. For comparison, we further define binding energy $E_b=-E_{ads}$, following which positive E_b means strong adsorption.

[1] P. Feng, W. Wang, K. Wang, S. Cheng, K. Jiang, Journal of Materials Chemistry A 2017, 5, 10261.

[2] J. Tan, J. Matz, P. Dong, J. Shen, M. Ye, Advanced Energy Materials 2021, 11, 2100046.

[3] I. D. R. Mackinnon, J. A. Alarco, P. C. T. J. Modeling, N. S. o. M. Science, 2013, 158.

[4] J. P. Perdew, J. A. Chevary, S. H. Vosko, K. A. Jackson, M. R. Pederson, D. J. Singh, C. Fiolhais, Physical Review B 1992, 46, 6671.

[5] B. Hammer, L. B. Hansen, J. K. Nørskov, Physical Review B 1999, 59, 7413; J. P. Perdew, K. Burke, M. Ernzerhof, Physical Review Letters 1996, 77, 3865.
